# The genome sequence of the Greater Wax Moth, *Galleria mellonella *(Linnaeus, 1758)

**DOI:** 10.12688/wellcomeopenres.21089.2

**Published:** 2025-10-10

**Authors:** Mark J. Sterling, Maxwell V. L. Barclay, David C. Lees, Natural History Museum Genome Acquisition Lab, Darwin Tree of Life Barcoding collective, Wellcome Sanger Institute Tree of Life Management, Samples and Laboratory team, Wellcome Sanger Institute Scientific Operations: Sequencing Operations, Wellcome Sanger Institute Tree of Life Core Informatics team, Tree of Life Core Informatics collective, Darwin Tree of Life Consortium

**Affiliations:** 1 Natural History Museum, London, England, UK

**Keywords:** Galleria mellonella, Greater Wax Moth, genome sequence, chromosomal, Lepidoptera

## Abstract

We present a genome assembly from an individual male *Galleria mellonella* (the Greater Wax Moth; Arthropoda; Insecta; Lepidoptera; Pyralidae). The genome sequence is 466.9 megabases in span. Most of the assembly is scaffolded into 30 chromosomal pseudomolecules, including the Z sex chromosome. Gene annotation of this assembly on Ensembl identified 14,075 protein-coding genes. The mitochondrial genome has also been assembled and is 15.37 kilobases in length. This assembly was generated as part of the Darwin Tree of Life project, which produces reference genomes for eukaryotic species found in Britain and Ireland.

## Species taxonomy

Eukaryota; Opisthokonta; Metazoa; Eumetazoa; Bilateria; Protostomia; Ecdysozoa; Panarthropoda; Arthropoda; Mandibulata; Pancrustacea; Hexapoda; Insecta; Dicondylia; Pterygota; Neoptera; Endopterygota; Amphiesmenoptera; Lepidoptera; Glossata; Neolepidoptera; Heteroneura; Ditrysia; Obtectomera; Pyraloidea; Pyralidae; Galleriinae; *Galleria*; *Galleria mellonella* (Linnaeus 1758) (NCBI:txid7137).

## Background

The Greater Wax Moth *Galleria mellonella* is a well-known pest of honey bees ( *Apis mellifera* Linnaeus, 1758 and *Apis cerana* Fabricius, 1793). Its larvae tunnel through honeycombs that contain honeybee larvae. The larval tunnels are lined with silk, which entangles emerging bees, thereby causing them to starve. The tunnels destroy combs, and honey is wasted as it leaks out when cell caps are eaten ( Kwadha *et al.*, 2017). However, *G. mellonella* is also beneficial. Its larva is used as a model for assessing the virulence of bacterial pathogens and the effectiveness of antimicrobial agents ( Pereira *et al.*, 2020) and it is mass produced for animal feed ( Van Huis & Tomberlin, 2017). Also, in 2017, after a technician breeding *G. mellonella* noticed holes in a plastic bag, Bombelli *et al.* (2017) found that the larvae could degrade polyethylene. Subsequent research ( Sanluis-Verdes *et al.*, 2022) has shown that enzymes contained in its larval saliva can oxidise and depolymerise polyethylene within hours at room temperature.

The Greater Wax Moth is a pyralid moth with a wingspan of 30–41 mm. It is sexually dimorphic. The female is usually larger than the male and the termen of the forewing of the male has a strong tornal lobe whereas in the female the tornal lobe is weak or absent. The forewing is brownish, sometimes shaded lighter, with a postmedial line comprising of a series of short dark streaks. A dark basal streak is usually present. The female is often suffused purplish brown, rendering the wing markings more obscure. In the UK, the moth flies from June to October, probably in two overlapping broods. It can be found in and around beehives, flying at night near the hives, and also comes to light in small numbers ( Goater *et al.*, 1986: 99; Parsons & Clancy, 2023: 47).

*G. mellonella* has a cosmopolitan distribution. It has been reported in twenty-seven African countries, nine Asian countries, four North American countries, three Latin American countries, Australasia, ten European countries, and five island countries ( Kwadha *et al.*, 2017), as well as in South America, Hawaii, and the Malagasy Region ( GBIF Secretariat, 2024). It is likely to occur wherever honeybees are present.

*G. mellonella* exhibits (25/10/2023) a single cosmopolitan cluster on BOLD (BIN, BOLD:AAA0965). However, there are two additional related clusters. The first of these (BOLD:ABA6806), between 4–5.5% divergent from it, is Asiatic (distributed from India, China and Korea to New Guinea). This cluster represents *G. similis* Roh & Song, 2020, and includes some apparent misidentifications as *G. mellonella*. The second cluster (BOLD:ACN5780), 3.69–3.85% divergent from either of the first two, and presently identified as *G. mellonella*, is known from Gabon. Roh *et al.* (2020) distinguished *G. similis* based on both on differences in morphology and in COI, Wingless and CAD genes. The Greater Wax Moth is a close relative of the Lesser Wax Moth, *Achroia grisella*, which is also the subject of genomic studies (e.g. Koseva *et al.*, 2019), and also consumes polyethylene ( Chalup *et al.*, 2018).

The genome of *G. mellonella* will be very useful for more detailed phylogenetic, biochemical and physiological studies. Previous genome sequences have been published, including those by Kong *et al.* (2019) and Lange *et al*. (2018). The current reference genome for *Galleria mellonella* is the assembly CSIRO_AGI_GalMel_v1 (GCA_026898425.1), submitted in 2022, which is a contig-level whole genome sequence. Here we present a chromosomally-complete genome sequence for *Galleria mellonella*, generated as part of the Darwin Tree of Life project. The specimen used was a single mature larva from a culture kept in London, England as food for pet reptiles.

## Genome sequence report

**Figure 1.**
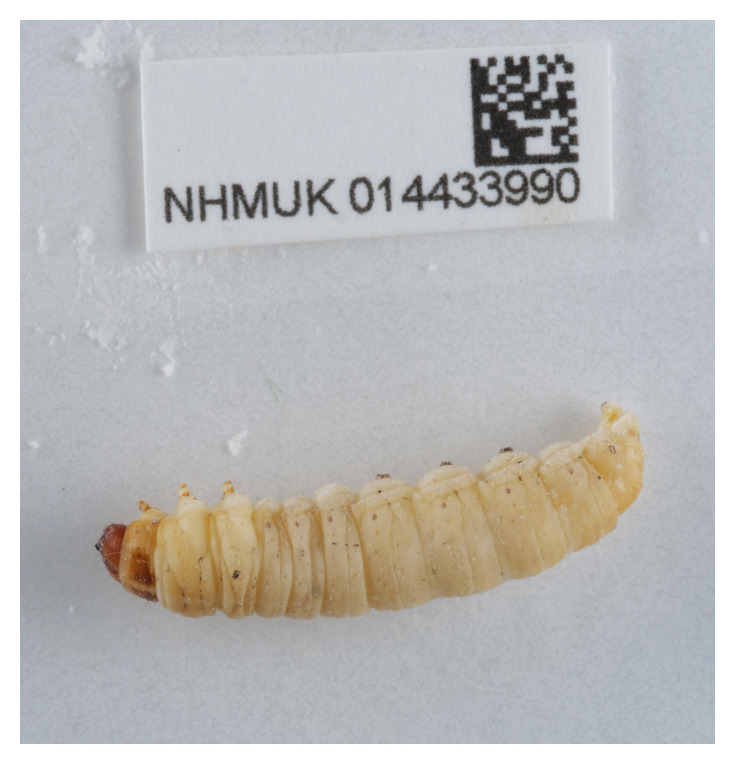
Photograph of the *Galleria mellonella* (ilGalMell1) specimen used for genome sequencing.

The genome was sequenced from a specimen of *Galleria mellonella* ( Figure 1). A total of 55-fold coverage in Pacific Biosciences single-molecule HiFi long reads was generated. Primary assembly contigs were scaffolded with chromosome conformation Hi-C data. Manual assembly curation corrected 6 missing joins or mis-joins and removed one haplotypic duplication.

**Figure 2.**
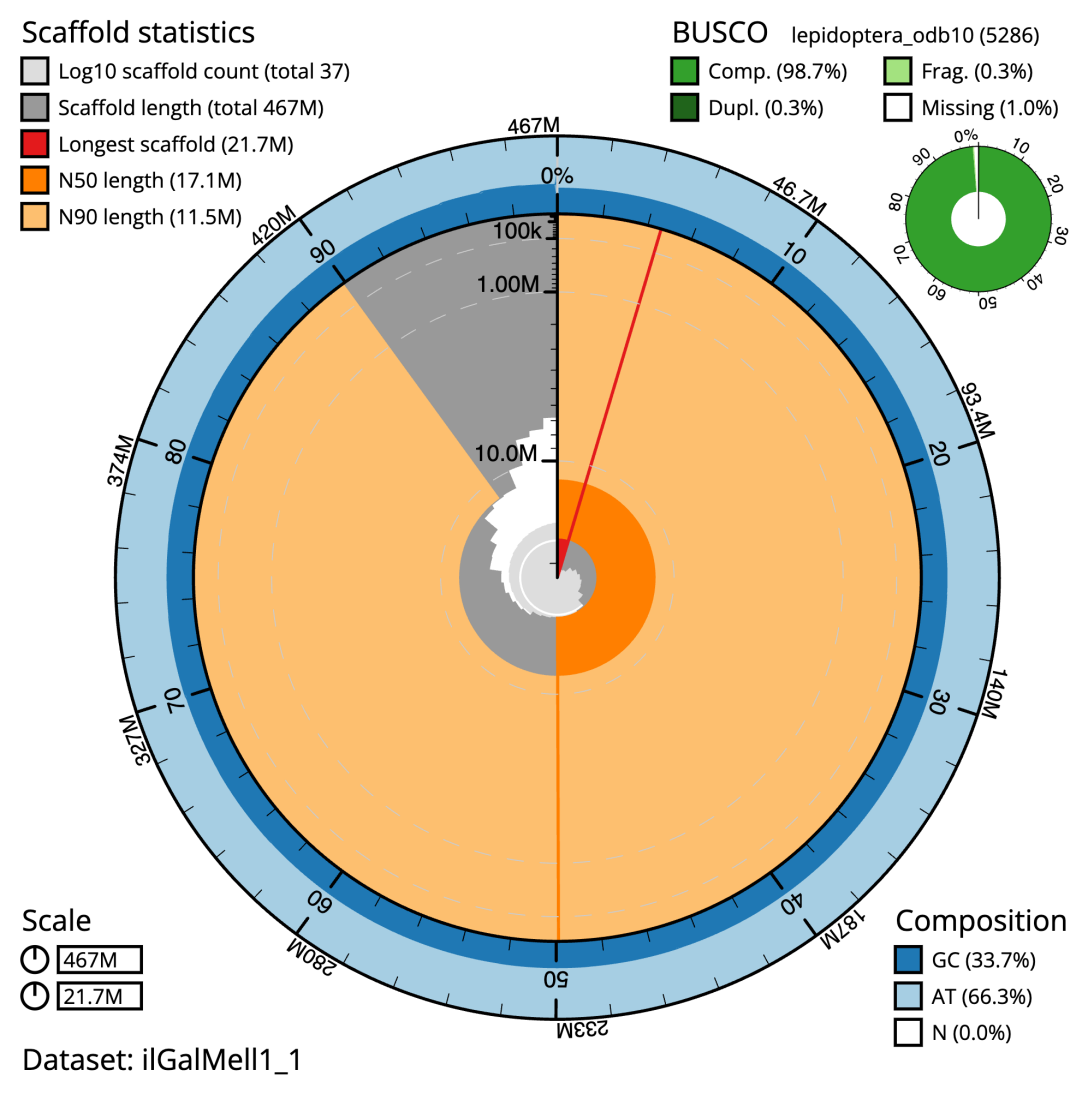
Genome assembly of *Galleria mellonella*, ilGalMell1.1: metrics. The BlobToolKit snail plot shows N50 metrics and BUSCO gene completeness. The main plot is divided into 1,000 size-ordered bins around the circumference with each bin representing 0.1% of the 466,922,119 bp assembly. The distribution of scaffold lengths is shown in dark grey with the plot radius scaled to the longest scaffold present in the assembly (21,666,304 bp, shown in red). Orange and pale-orange arcs show the N50 and N90 scaffold lengths (17,091,270 and 11,548,636 bp), respectively. The pale grey spiral shows the cumulative scaffold count on a log scale with white scale lines showing successive orders of magnitude. The blue and pale-blue area around the outside of the plot shows the distribution of GC, AT and N percentages in the same bins as the inner plot. A summary of complete, fragmented, duplicated and missing BUSCO genes in the lepidoptera_odb10 set is shown in the top right. An interactive version of this figure is available at https://blobtoolkit.genomehubs.org/view/ilGalMell1_1/dataset/ilGalMell1_1/snail.

**Figure 3.**
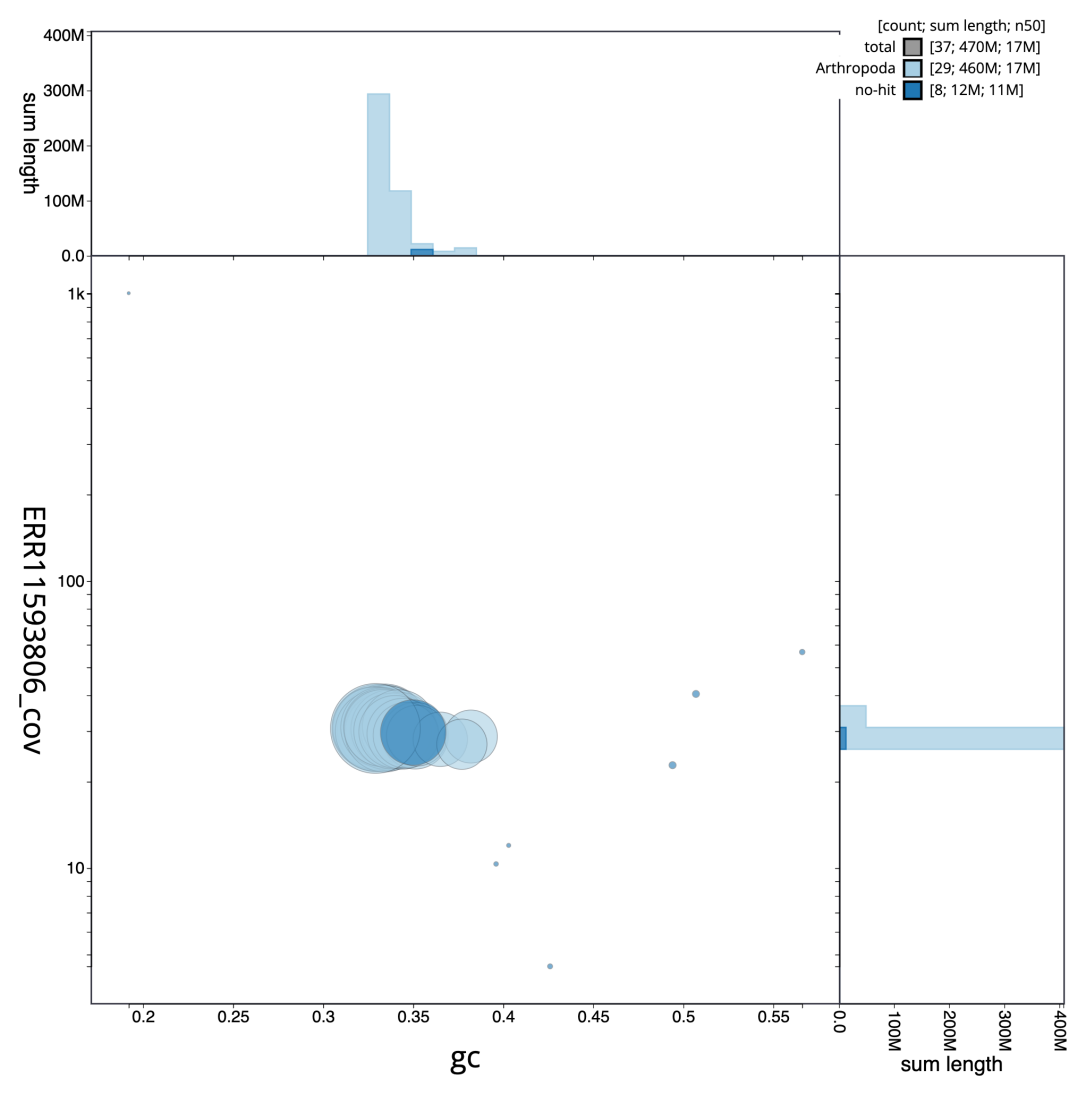
Genome assembly of *Galleria mellonella*, ilGalMell1.1: BlobToolKit GC-coverage plot. Sequences are coloured by phylum. Circles are sized in proportion to sequence length. Histograms show the distribution of sequence length sum along each axis. An interactive version of this figure is available at https://blobtoolkit.genomehubs.org/view/ilGalMell1_1/dataset/ilGalMell1_1/blob.

**Figure 4.**
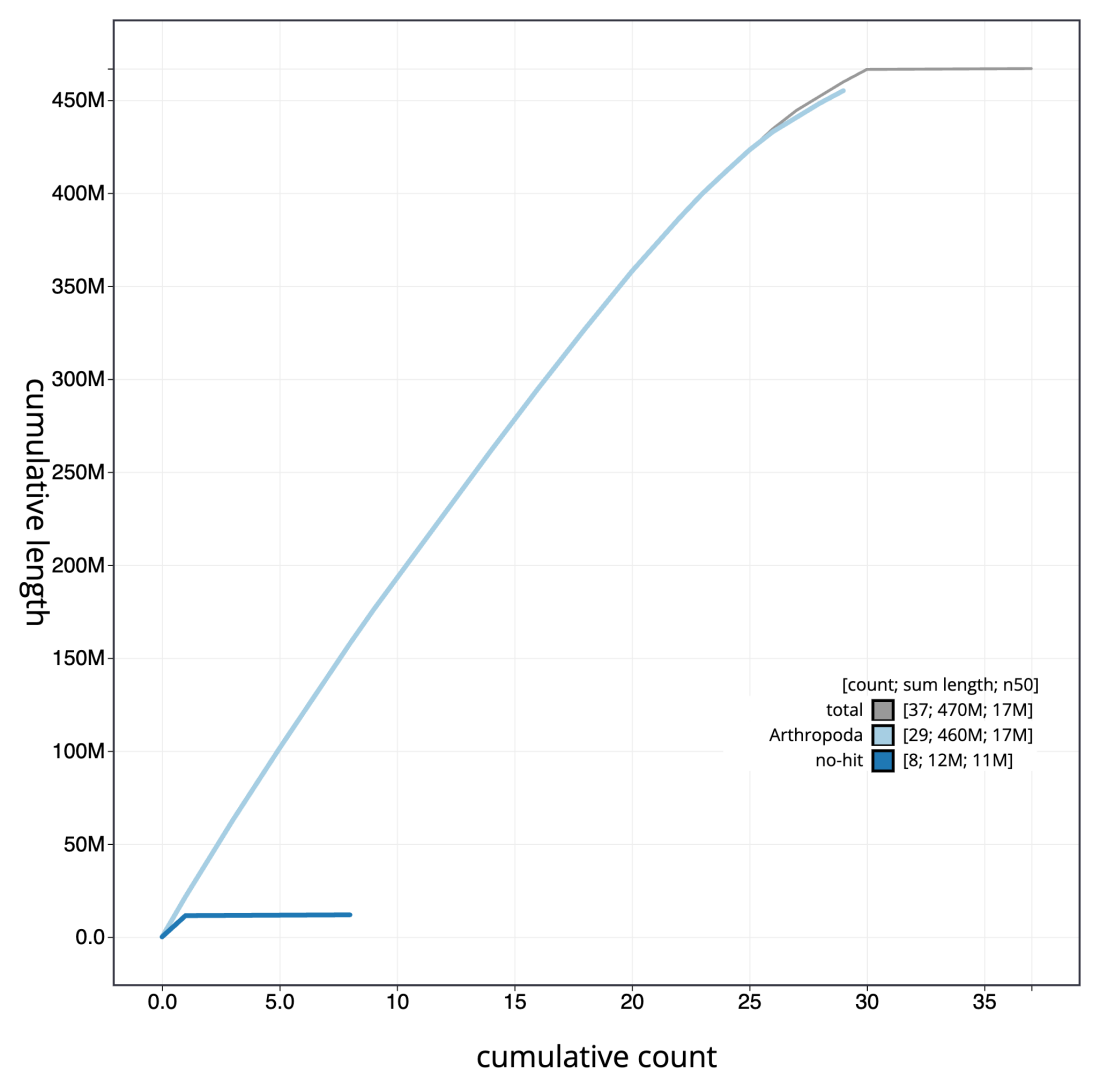
Genome assembly of *Galleria mellonella*, ilGalMell1.1: BlobToolKit cumulative sequence plot. The grey line shows cumulative length for all sequences. Coloured lines show cumulative lengths of sequences assigned to each phylum using the buscogenes taxrule. An interactive version of this figure is available at https://blobtoolkit.genomehubs.org/view/ilGalMell1_1/dataset/ilGalMell1_1/cumulative.

**Figure 5.**
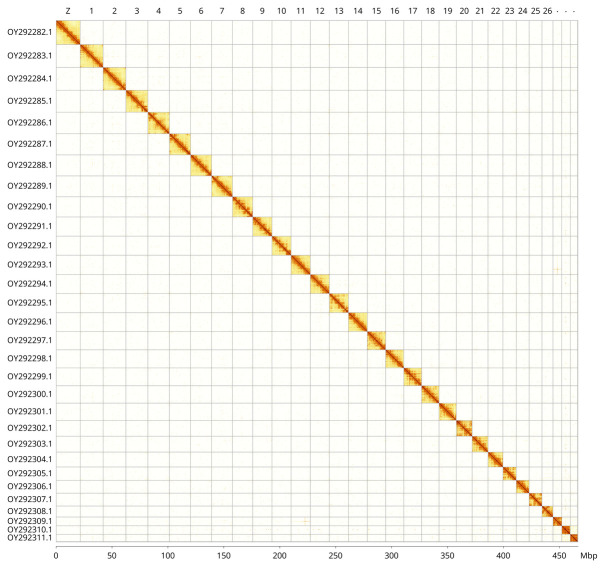
Genome assembly of *Galleria mellonella*, ilGalMell1.1: Hi-C contact map of the ilGalMell1.1 assembly, visualised using PretextView. Chromosomes are shown in order of size from left to right and top to bottom. An interactive version of this figure in HiGlass format may be viewed at https://genome-note-higlass.tol.sanger.ac.uk/l/?d=Qvk3ccsVSbCEKXKBH37T_w.

The final assembly has a total length of 466.9 Mb in 36 sequence scaffolds with a scaffold N50 of 17.1 Mb ( Table 1). The snail plot in Figure 2 provides a summary of the assembly statistics, while the distribution of assembly scaffolds on GC proportion and coverage is shown in Figure 3. The cumulative assembly plot in Figure 4 shows curves for subsets of scaffolds assigned to different phyla. Most (99.9%) of the assembly sequence was assigned to 30 chromosomal-level scaffolds, representing 29 autosomes and the Z sex chromosome. Chromosome-scale scaffolds confirmed by the Hi-C data are named in order of size ( Figure 5; Table 2). The Z chromosome identified based on synteny with *Pyralis farinalis* (GCA_947507595.1) ( Broad *et al.*, 2023). While not fully phased, the assembly deposited is of one haplotype. Contigs corresponding to the second haplotype have also been deposited. The mitochondrial genome was also assembled and can be found as a contig within the multifasta file of the genome submission.

**Table 1.** Genome data for *Galleria mellonella*, ilGalMell1.1.

Project accession data		
Assembly identifier	ilGalMell1.1	
Species	*Galleria mellonella*	
Specimen	ilGalMell1	
NCBI taxonomy ID	7137	
BioProject	PRJEB63442	
BioSample ID	SAMEA112222352	
Isolate information	ilGalMell1 larva: whole organism (DNA and Hi-C sequencing)	
Assembly metrics *		*Benchmark*
Consensus quality (QV)	Primary: 56.1; alternate: 54.9; combined: 55.3	*≥ 40*
*k*-mer completeness	Primary: 83.69%; alternate: 63.88%; combined: 99.54%	*≥ 95%*
BUSCO **	C:98.7%[S:98.5%,D:0.3%], F:0.3%,M:1.0%,n:5,286	*S > 90%; D < 5%*
Percentage of assembly mapped to chromosomes	99.9%	*≥ 90%*
Sex chromosomes	ZZ	*localised homologous pairs*
Organelles	Mitochondrial genome: 15.37 kb	*complete single alleles*
Raw data accessions		
PacificBiosciences SEQUEL II	ERR11593806	
Hi-C Illumina	ERR11606321	
Genome assembly		
Assembly accession	GCA_958496185.1	
*Accession of alternate haplotype*	GCA_958496355.1	
Span (Mb)	466.9	
Number of contigs	98	
Contig N50 length (Mb)	9.3	
Number of scaffolds	36	
Scaffold N50 length (Mb)	17.1	
Longest scaffold (Mb)	21.67	

* Assembly metric benchmarks are adapted from column VGP-2020 of “Table 1: Proposed standards and metrics for defining genome assembly quality” from Rhie *et al.* (2021). ** BUSCO scores based on the lepidoptera_odb10 BUSCO set using version 5.3.2. C = complete [S = single copy, D = duplicated], F = fragmented, M = missing, n = number of orthologues in comparison. A full set of BUSCO scores is available at https://blobtoolkit.genomehubs.org/view/ilGalMell1_1/dataset/ilGalMell1_1/busco.

**Table 2.** Chromosomal pseudomolecules in the genome assembly of *Galleria mellonella*, ilGalMell1.

INSDC accession	Chromosome	Length (Mb)	GC%
OY292283.1	1	20.5	33.0
OY292284.1	2	20.38	33.5
OY292285.1	3	19.66	33.5
OY292286.1	4	19.22	33.5
OY292287.1	5	18.95	33.0
OY292288.1	6	18.75	33.0
OY292289.1	7	18.69	33.0
OY292290.1	8	18.05	33.0
OY292291.1	9	17.26	33.0
OY292292.1	10	17.16	33.0
OY292293.1	11	17.11	33.5
OY292294.1	12	17.09	33.5
OY292295.1	13	17.0	33.5
OY292296.1	14	16.86	33.5
OY292297.1	15	16.41	33.5
OY292298.1	16	16.21	33.5
OY292299.1	17	15.95	33.5
OY292300.1	18	15.62	34.0
OY292301.1	19	15.39	34.0
OY292302.1	20	14.22	34.0
OY292303.1	21	14.13	34.0
OY292304.1	22	13.42	34.5
OY292305.1	23	11.86	35.0
OY292306.1	24	11.55	34.5
OY292307.1	25	11.44	35.0
OY292308.1	26	9.85	35.0
OY292309.1	27	7.88	36.5
OY292310.1	28	7.47	38.0
OY292311.1	29	6.73	37.5
OY292282.1	Z	21.67	33.0
OY292312.1	MT	0.02	19.5

The estimated Quality Value (QV) of the final assembly is 56.1 with *k*-mer completeness of 99.99%, and the assembly has a BUSCO v5.3.2 completeness of 98.7% (single = 98.5%, duplicated = 0.3%), using the lepidoptera_odb10 reference set ( *n* = 5,286).

Metadata for specimens, barcode results, spectra estimates, sequencing runs, contaminants and pre-curation assembly statistics are given at https://links.tol.sanger.ac.uk/species/7137.

## Genome annotation report

The *Galleria mellonella* genome assembly (GCA_958496185.1) was annotated by Ensembl at the European Bioinformatics Institute (EBI). This annotation includes 43,507 transcribed mRNAs from 14,075 protein-coding and 10,791 non-coding genes. The average transcript length is 17,527.42 bp, with an average of 1.75 coding transcripts per gene and 6.21 exons per transcript. For further information about the annotation, please refer to the annotation page on Ensembl.

## Methods

### Sample acquisition and nucleic acid extraction

A mature larva of *Galleria mellonella* (specimen ID NHMUK014433990, ToLID ilGalMell1) was collected from a laboratory culture kept in Fulham, London, UK, on 2021-11-25. The specimen was collected and identified by Maxwell Barclay (Natural History Museum) and dry-frozen at –80 °C.

The workflow for high molecular weight (HMW) DNA extraction at the Wellcome Sanger Institute (WSI) includes a sequence of core procedures: sample preparation; sample homogenisation, DNA extraction, fragmentation, and clean-up. In sample preparation, the ilGalMell1 sample was weighed and dissected on dry ice ( Jay *et al.*, 2023). Tissue from the whole organism was homogenised using a PowerMasher II tissue disruptor ( Denton *et al.*, 2023a).

HMW DNA was extracted in the WSI Scientific Operations core using the Automated MagAttract v2 protocol ( Oatley *et al.*, 2023). The DNA was sheared into an average fragment size of 12–20 kb in a Megaruptor 3 system with speed setting 31 ( Bates *et al.*, 2023). Sheared DNA was purified by solid-phase reversible immobilisation ( Strickland *et al.*, 2023): in brief, the method employs a 1.8X ratio of AMPure PB beads to sample to eliminate shorter fragments and concentrate the DNA. The concentration of the sheared and purified DNA was assessed using a Nanodrop spectrophotometer and Qubit Fluorometer and Qubit dsDNA High Sensitivity Assay kit. Fragment size distribution was evaluated by running the sample on the FemtoPulse system.

Protocols developed by the WSI Tree of Life laboratory are publicly available on protocols.io ( Denton *et al.*, 2023b).

### Sequencing

Pacific Biosciences HiFi circular consensus DNA sequencing libraries were constructed according to the manufacturers’ instructions. DNA sequencing was performed by the Scientific Operations core at the WSI on a Pacific Biosciences SEQUEL II instrument. Hi-C data were also generated from remaining tissue of ilGalMell1 using the Arima2 kit and sequenced on the Illumina NovaSeq 6000 instrument.

### Genome assembly, curation and evaluation

Assembly was carried out with Hifiasm ( Cheng *et al.*, 2021) using the primary option. Haplotypic duplication was identified and removed with purge_dups ( Guan *et al.*, 2020). The assembly was then scaffolded with Hi-C data ( Rao *et al.*, 2014) using YaHS ( Zhou *et al.*, 2023). The assembly was checked for contamination and corrected as described previously ( Howe *et al.*, 2021). Manual curation was performed using HiGlass ( Kerpedjiev *et al.*, 2018) and PretextView ( Harry, 2022). The mitochondrial genome was assembled using MitoHiFi ( Uliano-Silva *et al.*, 2023), which runs MitoFinder ( Allio *et al.*, 2020) and uses these annotations to select the final mitochondrial contig and to ensure the general quality of the sequence.

A Hi-C map for the final assembly was produced using bwa-mem2 ( Vasimuddin *et al.*, 2019) in the Cooler file format ( Abdennur & Mirny, 2020). To assess the assembly metrics, the *k*-mer completeness and QV consensus quality values were calculated in Merqury.FK ( Rhie *et al.*, 2020). The genome was analysed within the BlobToolKit environment ( Challis *et al.*, 2020) and BUSCO scores ( Manni *et al.*, 2021; Simão *et al.*, 2015) were calculated.

Table 3 contains a list of relevant software tool versions and sources.

**Table 3.** Software tools: versions and sources.

Software tool	Version	Source
BlobToolKit	4.2.1	https://github.com/blobtoolkit/blobtoolkit
BUSCO	5.3.2	https://gitlab.com/ezlab/busco
Hifiasm	0.19.5-r587	https://github.com/chhylp123/hifiasm
HiGlass	1.11.6	https://github.com/higlass/higlass
Merqury	1.1.2	https://github.com/thegenemyers/MERQURY.FK
MitoHiFi	3	https://github.com/marcelauliano/MitoHiFi
PretextView	0.2	https://github.com/wtsi-hpag/PretextView
purge_dups	1.2.5	https://github.com/dfguan/purge_dups
YaHS	1.2a.2	https://github.com/c-zhou/yahs

### Wellcome Sanger Institute – Legal and Governance

The materials that have contributed to this genome note have been supplied by a Darwin Tree of Life Partner. The submission of materials by a Darwin Tree of Life Partner is subject to the **‘Darwin Tree of Life Project Sampling Code of Practice’**, which can be found in full on the Darwin Tree of Life website here. By agreeing with and signing up to the Sampling Code of Practice, the Darwin Tree of Life Partner agrees they will meet the legal and ethical requirements and standards set out within this document in respect of all samples acquired for, and supplied to, the Darwin Tree of Life Project. 

Further, the Wellcome Sanger Institute employs a process whereby due diligence is carried out proportionate to the nature of the materials themselves, and the circumstances under which they have been/are to be collected and provided for use. The purpose of this is to address and mitigate any potential legal and/or ethical implications of receipt and use of the materials as part of the research project, and to ensure that in doing so we align with best practice wherever possible. The overarching areas of consideration are:

• Ethical review of provenance and sourcing of the material

• Legality of collection, transfer and use (national and international)

Each transfer of samples is further undertaken according to a Research Collaboration Agreement or Material Transfer Agreement entered into by the Darwin Tree of Life Partner, Genome Research Limited (operating as the Wellcome Sanger Institute), and in some circumstances other Darwin Tree of Life collaborators.

## Data Availability

European Nucleotide Archive: *Galleria mellonella* (greater wax moth). Accession number PRJEB63442; https://identifiers.org/ena.embl/PRJEB63442 ( Wellcome Sanger Institute, 2023). The genome sequence is released openly for reuse. The *Galleria mellonella* genome sequencing initiative is part of the Darwin Tree of Life (DToL) project. All raw sequence data and the assembly have been deposited in INSDC databases. The genome will be annotated using available RNA-Seq data and presented through the Ensembl pipeline at the European Bioinformatics Institute. Raw data and assembly accession identifiers are reported in Table 1.

## Author information

Members of the Natural History Museum Genome Acquisition Lab are listed here: https://doi.org/10.5281/zenodo.7139035.

Members of the Darwin Tree of Life Barcoding collective are listed here: https://doi.org/10.5281/zenodo.4893703.

Members of the Wellcome Sanger Institute Tree of Life Management, Samples and Laboratory team are listed here: https://doi.org/10.5281/zenodo.10066175.

Members of Wellcome Sanger Institute Scientific Operations: Sequencing Operations are listed here: https://doi.org/10.5281/zenodo.10043364.

Members of the Wellcome Sanger Institute Tree of Life Core Informatics team are listed here: https://doi.org/10.5281/zenodo.10066637.

Members of the Tree of Life Core Informatics collective are listed here: https://doi.org/10.5281/zenodo.5013541.

Members of the Darwin Tree of Life Consortium are listed here: https://doi.org/10.5281/zenodo.4783558.
