# Supplementary material for: The genome sequence of the Greater Wax Moth, Galleria mellonella (Linnaeus, 1758)
Source: Wellcome Open Res. 2025 Oct 10;9:101. Originally published 2024 Mar 1. [Version 2] doi: 10.12688/wellcomeopenres.21089.2 (PMC12605590; doi:10.12688/wellcomeopenres.21089.2)
Supplement: Peer review history

# Reviewer report: Reviewer response for version 2

**DOI:** 10.21956/wellcomeopenres.27543.r136106

**Published:** 2025-11-20

**Authors:** Wai Lok So

**Affiliations:** 1 The Chinese University of Hong Kong, Hong Kong, China

The authors generated a chromosomal level genome of an ecological and economical important moth species, *Galleria mellonella*. The genome was assebmled in 30 chromosomal-level scaffolds with the sex chromosome being identified. The sequencing, assembly and annotation methods were scientifically sound and logical. The genome is believed to be benefitial to different communities in different resarch fields, including ecology, genetics and evolution. 

 There are some minor comments regarding on the manuscript, as stated below:

 1. Please kindly state if the sex of the sample collected can be identified. And if possible, kindly add a scale bar to the figure in Figure 1.

 2. As stated in the method part, the sequencing sample was collected from a laboratory culture, menawhile the authors also reported that there are several population clusters globally in the backgroudn section. Is that possible that the authors can share which cluster this species is belonging to? This information will be useful to further research when this genome is being used.

 3. The authors reported that the final assembly consists of 36 sequence scaffolds, but the resulting genome consists of only 30 chromosomal-level scaffolds. Please kindly indicate what are the rest of the 6 scaffolds. Are they repetitive sequences that could not be nicely assembled, or they cannot be annotated?

Are sufficient details of methods and materials provided to allow replication by others?

Yes

Is the rationale for creating the dataset(s) clearly described?

Yes

Are the datasets clearly presented in a useable and accessible format?

Yes

Are the protocols appropriate and is the work technically sound?

Yes

Reviewer Expertise:

genomics, genetics, evolution, arthropods, myriapods

I confirm that I have read this submission and believe that I have an appropriate level of expertise to confirm that it is of an acceptable scientific standard.

# Reviewer report: Reviewer response for version 2

**DOI:** 10.21956/wellcomeopenres.27543.r135738

**Published:** 2025-11-11

**Authors:** Christopher B. Cunningham

**Affiliations:** 1 Department of Entomology, University of Georgia, Athens, Georgia, USA

The revision addressed most of the comments. The annotation assessment is not fully reported, but at least the reader has a clear link to where to go to understand it. 

Are sufficient details of methods and materials provided to allow replication by others?

Partly

Is the rationale for creating the dataset(s) clearly described?

No

Are the datasets clearly presented in a useable and accessible format?

Yes

Are the protocols appropriate and is the work technically sound?

Yes

Reviewer Expertise:

Genomics, genetics, epigenetics, behavior, insects.

I confirm that I have read this submission and believe that I have an appropriate level of expertise to confirm that it is of an acceptable scientific standard.

# Reviewer report: Reviewer response for version 1

**DOI:** 10.21956/wellcomeopenres.23329.r92096

**Published:** 2024-08-26

**Authors:** Christopher B. Cunningham

**Affiliations:** 1 Department of Entomology, University of Georgia, Athens, Georgia, USA

I think this is a good resource for the insect genomics community. The methods are sound and relatively well documented. The article needs a few more details to be reasonably reproducible. The genome assembly itself is clearly of high quality.

**Abstract**

 There is no justification within the abstract, just results. At least a sentence that this was part of a large survey of insects is needed. And one sentence for its possible value.

**Keywords**

 Not present.  

**Background**

 The background contains lots of scientifically irrelevant material. An antidote is fine, but this is beyond that. The manuscript acknowledges previous genome assemblies, but does not state how it improves upon those other than continuity; greater justification of this this work is needed, is it more gene complete, does it contain more sequence, etc. Without an quality-controlled annotation, the new assembly is of lesser value. It is also referred to by two different common names, worm and moth. This species has also greatly helped us better understand insect immunology itself, not just that it can be used as a model for vertebrates diseases.

 Figure 2/3/4/5 titles. These should be informative and tell the reader what you would like them to understand about the data; e.g., Fig 3. Little contamination was found in the final genome assembly.

 Figure 4. has very little information and can easily be summarized as a sentence in the results. The same is true of Fig 5

 All parameter setting if they were different from default of each piece of bioinformatic software needs to be specified. Just put a sentence to begin that everything was default unless specified otherwise. What assessment was done to ensure that default was adequate? This is a non-trivial issue.

 Table 3 is of little to no value and contains much duplicated information. Just add the version numbers of software used inline at the appropriate places in the methods section.

 Is the Ethics statement needed? It just says the provider of the sample should meet some agreed upon standard, but does not actually say that they did in this case. Either drop the statement or actually say the standard was met for this sample. This is not a paper about the standards of collection.

 Add something to the report that contextualize the research for the reader how this new resource improves upon what is already known. This is important for the reader to know that this does not contain as much information as previous assemblies because it lacks an annotation. Do the authors believe that this work can contribute to genome structure evolution, etc? This does not to be extensive or highly directed, but there is currently no justification of this work outside of the Data Availability Statement. Even Data Note should contain rationale for the work.

Are sufficient details of methods and materials provided to allow replication by others?

Partly

Is the rationale for creating the dataset(s) clearly described?

No

Are the datasets clearly presented in a useable and accessible format?

Yes

Are the protocols appropriate and is the work technically sound?

Yes

Reviewer Expertise:

Genomics, genetics, epigenetics, behavior, insects.

I confirm that I have read this submission and believe that I have an appropriate level of expertise to confirm that it is of an acceptable scientific standard, however I have significant reservations, as outlined above.

# Reviewer report: Reviewer response for version 1

**DOI:** 10.21956/wellcomeopenres.23329.r90651

**Published:** 2024-08-14

**Authors:** Maurijn van der Zee

**Affiliations:** 1 Institute of Biology, Leiden University, Sylviusweg, Leiden, The Netherlands

Sterling *et al*. present here the genome sequence of the wax moth Galleria mellonella. The assembly is of high quality, particularly because Hi-C data have been used to scaffold the contigs into complete chromosomes. As the authors note, genomes of this moth have been published before (Kong *et al.* 2019; Lange *et al.* 2018) but contain many contigs. Likely crossing during the publication process, another improved reference was presented by Australian researchers Young *et al. (*2024 ^1^ ).  I think it would be good if Sterling *et al.* include a statement how their dataset relates to the recent genome by Young *et al.* This does not seem a major issue to me, as the Young *et al*. genome seems to consist, in my understanding, of 221 contigs that have not been scaffolded at chromosome-level.

Are sufficient details of methods and materials provided to allow replication by others?

Yes

Is the rationale for creating the dataset(s) clearly described?

Yes

Are the datasets clearly presented in a useable and accessible format?

Yes

Are the protocols appropriate and is the work technically sound?

Yes

Reviewer Expertise:

Comparative genomics, evo-devo, life histories

I confirm that I have read this submission and believe that I have an appropriate level of expertise to confirm that it is of an acceptable scientific standard.

## References

[1] : Improved reference quality genome sequence of the plastic-degrading greater wax moth, Galleria mellonella. *G3 (Bethesda)* .2024;14(6) : 10.1093/g3journal/jkae070 10.1093/g3journal/jkae070 38564250 PMC11152082
